# An *ent*-kaurane diterpenoid from *Isodon japonica* var. glaucocalyx

**DOI:** 10.1107/S1600536809027159

**Published:** 2009-07-18

**Authors:** Su-Ping Bai, Guo-Sheng Luo, Xiao-Yi Zhang, Wei Liu

**Affiliations:** a School of Pharmacy, Xinxiang Medical University, Xinxiang, Henan 453003, People’s Republic of China; b Department of Science and Technology, Xinxiang Medical University, Xinxiang, Henan 453003, People’s Republic of China

## Abstract

The title compound, 14β-acet­oxy-7α-hydr­oxy-*ent*-kaur-16-ene-3,15-dione or glaucocalyxin B, C_22_H_30_O_5_, a natural *ent*-kaurane diterpenoid, is composed of four rings with the expected *cis* and *trans* ring junctions. In the crystal structure, there are two mol­ecules in the asymmetric unit related by a noncrystallographic twofold screw axis, and ring *A* is disordered [ratio occupancies 0.829 (19):0.171 (19)], such that both chair and boat conformations are present, but with the boat conformation as the major component. In the crystal, mol­ecules are linked by inter­molecular O—H⋯O hydrogen bonds.

## Related literature

For related literature on the genus *Isodon* and diterpenoids therefrom, see: Liu *et al.* (1988 ▶); Kim *et al.* (1992 ▶); Sun *et al.* (2001 ▶); Bai *et al.* (2005 ▶). For expected bond-length ranges, see: Allen *et al.* (1987 ▶). 
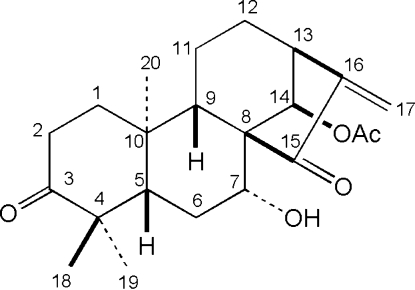


## Experimental

### 

#### Crystal data

C_22_H_30_O_5_
*M* *_r_* = 374.46
Monoclinic, 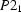

*a* = 8.485 (4) Å
*b* = 23.786 (10) Å
*c* = 9.930 (4) Å
β = 91.039 (17)°
*V* = 2003.8 (15) Å^3^
*Z* = 4
Mo *K*α radiation
μ = 0.09 mm^−1^
*T* = 296 K
0.36 × 0.34 × 0.32 mm

#### Data collection

Rigaku R-AXIS RAPID diffractometer
Absorption correction: none
19620 measured reflections
4669 independent reflections
2856 reflections with *I* > 2σ(*I*)
*R*_int_ = 0.070

#### Refinement

*R*[*F* ^2^ > 2σ(*F* ^2^)] = 0.063
*wR*(*F* ^2^) = 0.164
*S* = 1.00
4669 reflections
524 parameters
14 restraints
H-atom parameters constrained
Δρ_max_ = 0.19 e Å^−3^
Δρ_min_ = −0.20 e Å^−3^

### 

Data collection: *RAPID-AUTO* (Rigaku, 2004 ▶); cell refinement: *RAPID-AUTO*; data reduction: *RAPID-AUTO*; program(s) used to solve structure: *SHELXS97* (Sheldrick, 2008 ▶); program(s) used to refine structure: *SHELXL97* (Sheldrick, 2008 ▶); molecular graphics: *SHELXTL* (Sheldrick, 2008 ▶); software used to prepare material for publication: *SHELXTL*.

## Supplementary Material

Crystal structure: contains datablocks global, I. DOI: 10.1107/S1600536809027159/ez2171sup1.cif

Structure factors: contains datablocks I. DOI: 10.1107/S1600536809027159/ez2171Isup2.hkl

Additional supplementary materials:  crystallographic information; 3D view; checkCIF report

## Figures and Tables

**Table 1:** Hydrogen-bond geometry (Å, °)

*D*—H⋯*A*	*D*—H	H⋯*A*	*D*⋯*A*	*D*—H⋯*A*
O2—H2O⋯O1^i^	0.82	2.09	2.747 (6)	137
O2—H2O⋯O1^i^	0.82	2.09	2.747 (6)	137
O2′—H2O′⋯O1′^i^	0.82	2.05	2.764 (5)	146
O2′—H2O′⋯O1*^i^	0.82	2.24	2.89 (3)	136

Symmetry code: (i) 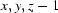.

**Fig. 1.**
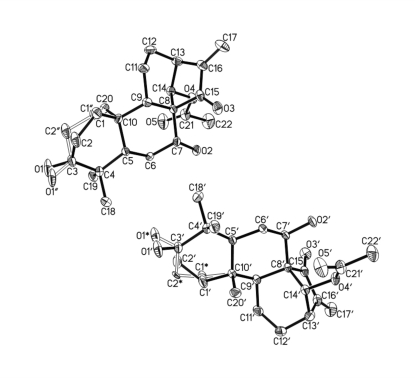
A view of the molecular structure of compound (I). Displacement ellipsoids are drawn at the 50% probability level.

**Fig. 2.**
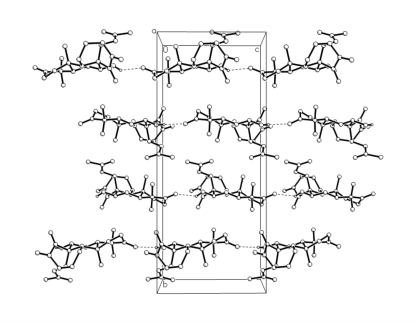
The crystal packing of (I), viewed along the b axis, showing the O—H···O hydrogen bonds as dashed lines.

## supplementary crystallographic information

### Comment

The title compound (I) is a natural *ent*-kaurane diterpenoid isolated from the medicinal plant Isodon japonica var. glaucocalyx. This plant has been used in antibacterial, inflammation-diminishing and stomachic agents. The structure has been postulated previously based on spectroscopic methods (Liu *et al.*, 1988; Kim *et al.*,1992; Bai *et al.*, 2005). In order to further confirm the structure and conformation of (I), a crystal structure analysis, reported here, was undertaken.

The X-ray crystallographic analysis of (I) confirms the previously proposed molecular structure of (I). Fig. 1 shows its conformation: two carbonyl groups located at C3 and C15, while a hydroxyl group and an acetoxyl group adopt α and β-orientations at C7 and C14 respectively. There is a *trans* junction between ring A (C1–C5/C10) and ring B (C5–C10); *cis* junctions are present between ring B and ring C (C8/C9/C11–C14), and ring C and ring D (C8/C13–C16).

The crystal structure analysis shows that there are two molecules in the asymmetric unit, each with different bond lengths and angles, but which are within expected ranges (Allen *et al.*, 1987). In both molecules ring B adopts a chair conformation and ring C has a slight-twist chair conformation. Ring A is disordered, such that both chair and boat conformations are present, but with the boat conformation as the major component. The ratios of boat to chair conformations are 66.7%:32.3% for C1–C5/C10, and 82.9%:17.1% for C1'–C5'/C10'. Ring D shows an envelope conformation; the flap atom, C14, lies 0.660 (6) Å from the plane defined by atoms C8, C15, C16 and C13 [0.665 (7) Å for atom C14'].

Compound (I) contains seven chiral centers at C5(S), C7(*R*), C8(*R*), C9(S), C10(*R*), C13(*R*) and C14(*R*). Although the absolute configuration could not be reliably determined from anomalous dispersion effects, the negative optical rotation showed this compound to be in the *ent*-kaurane seuies as reported in genus Isodon (Sun *et al.*, 2001), rather than in the kaurane series, and so allowed us to assign the correct configuration. In the crystal structure, the molecules are linked by O—H···O hydrogen bonds into chains parallel to the *c* axis (Table 1 and Fig. 2).

### Experimental

The dried and crushed leaves of Isodon japonica var. glaucocalyx (10 kg, collected from Hui Prefecture, Henan Province, China) were extracted four times with Me_2_CO/H_2_O (7:3, *v*/*v*) at room temperature over a period of seven days. The extract was filtered and the solvent was removed under reduced pressure. The residue was then partitioned between water and AcOEt. After removal of the solvent, the AcOEt residue was separated by repeated silica gel (200–300 mesh) column chromatography and recrystallization from CHCl_3_/Me_2_CO(20:1), giving 700 mg of compound (I) (m.p. 463–465 K. Optical rotation: [α]_D_^20^ -130 ° (c 0.95, CHCl_3_). Crystals suitable for X-ray analysis were obtained by slow evaporation of a solution of the compound (I) in Me_2_CO at room temperature.

### Refinement

All H atoms were included in calculated positions and refined as riding atoms, with C—H = 0.96 Å (CH_3_), 0.93 and 0.97 Å (CH_2_), and 0.98 Å (CH), and with *U*_iso_(H) = 1.2 *U*_eq_(C). In the absence of significant anomalous scattering effects, Friedel pairs were merged. The choice of enantiomer was based on comparison of the optical rotation with that of related compounds with known stereochemistry. Disorder in ring A was identified by peaks on a difference Fourier map. Each group of disordered atoms was refined with common site occupancies, and equivalent atoms were constrained to have the same anisotropic displacement parameters. The bond lengths in the disorder groups were restrained to values of 1.210 (3) Å (for all four C═O distances), 1.540 (3) Å (C1—C10, C1—C2, and equivalents) and 1.460 (3) Å (C2'—C3').

### Crystal data

**Table:**

C_22_H_30_O_5_	*D*_x_ = 1.241 Mg m^−^^3^
*M**_r_* = 374.46	Melting point: 463 K
Monoclinic, *P*2_1_	Mo *K*α radiation, λ = 0.71073 Å
*a* = 8.485 (4) Å	Cell parameters from 12776 reflections
*b* = 23.786 (10) Å	θ = 3.1–27.6°
*c* = 9.930 (4) Å	µ = 0.09 mm^−^^1^
β = 91.039 (17)°	*T* = 296 K
*V* = 2003.8 (15) Å^3^	Block, colourless
*Z* = 4	0.36 × 0.34 × 0.32 mm
*F*(000) = 808	

### Data collection

**Table:**

Rigaku R-AXIS RAPID diffractometer	2856 reflections with *I* > 2σ(*I*)
Radiation source: Rotating Anode	*R*_int_ = 0.070
graphite	θ_max_ = 27.5°, θ_min_ = 3.1°
ω scans	*h* = −10→11
19620 measured reflections	*k* = −30→28
4669 independent reflections	*l* = −12→12

### Refinement

**Table:**

Refinement on *F*^2^	Primary atom site location: structure-invariant direct methods
Least-squares matrix: full	Secondary atom site location: difference Fourier map
*R*[*F*^2^ > 2σ(*F*^2^)] = 0.063	Hydrogen site location: inferred from neighbouring sites
*wR*(*F*^2^) = 0.164	H-atom parameters constrained
*S* = 1.00	*w* = 1/[σ^2^(*F*_o_^2^) + (0.0896*P*)^2^ + 0.022*P*] where *P* = (*F*_o_^2^ + 2*F*_c_^2^)/3
4669 reflections	(Δ/σ)_max_ = 0.001
524 parameters	Δρ_max_ = 0.19 e Å^−^^3^
14 restraints	Δρ_min_ = −0.20 e Å^−^^3^

### Special details

**Table:**

Experimental. The assignment of absolute structure was based on comparison of the optical rotation with that of related compounds with known stereochemistry.
Geometry. All e.s.d.'s (except the e.s.d. in the dihedral angle between two l.s. planes) are estimated using the full covariance matrix. The cell e.s.d.'s are taken into account individually in the estimation of e.s.d.'s in distances, angles and torsion angles; correlations between e.s.d.'s in cell parameters are only used when they are defined by crystal symmetry. An approximate (isotropic) treatment of cell e.s.d.'s is used for estimating e.s.d.'s involving l.s. planes.
Refinement. Refinement of *F*^2^ against ALL reflections. The weighted *R*-factor *wR* and goodness of fit *S* are based on *F*^2^, conventional *R*-factors *R* are based on *F*, with *F* set to zero for negative *F*^2^. The threshold expression of *F*^2^ > σ(*F*^2^) is used only for calculating *R*-factors(gt) *etc*. and is not relevant to the choice of reflections for refinement. *R*-factors based on *F*^2^ are statistically about twice as large as those based on *F*, and *R*- factors based on ALL data will be even larger.

### Fractional atomic coordinates and isotropic or equivalent isotropic displacement parameters (Å^2^)

**Table:**

	*x*	*y*	*z*	*U*_iso_*/*U*_eq_	Occ. (<1)
O2	0.1468 (4)	0.62378 (17)	0.4126 (3)	0.0622 (9)	
H2O	0.1223	0.6421	0.3455	0.075*	
O3	−0.1799 (5)	0.69449 (15)	0.3591 (3)	0.0678 (10)	
O4	−0.0788 (4)	0.53978 (13)	0.3705 (3)	0.0528 (8)	
O5	0.1013 (6)	0.4948 (2)	0.4969 (5)	0.1003 (17)	
O1	0.1366 (10)	0.6324 (4)	1.1368 (5)	0.097 (3)	0.677 (9)
C1	−0.1799 (14)	0.6575 (9)	0.9035 (12)	0.0647 (16)	0.677 (9)
H1A	−0.2358	0.6891	0.8638	0.078*	0.677 (9)
H1B	−0.2575	0.6331	0.9438	0.078*	0.677 (9)
C2	−0.0687 (12)	0.6796 (5)	1.0157 (8)	0.077 (3)	0.677 (9)
H2A	−0.0502	0.7193	0.9995	0.092*	0.677 (9)
H2B	−0.1227	0.6766	1.1007	0.092*	0.677 (9)
O1"	0.1487 (19)	0.6698 (7)	1.1325 (10)	0.097 (3)	0.323 (9)
C1"	−0.173 (3)	0.6548 (19)	0.9108 (18)	0.0647 (16)	0.323 (9)
H1C	−0.1763	0.6949	0.8932	0.078*	0.323 (9)
H1D	−0.2805	0.6418	0.9200	0.078*	0.323 (9)
C2"	−0.082 (3)	0.6443 (12)	1.0439 (18)	0.077 (3)	0.323 (9)
H2C	−0.1191	0.6701	1.1117	0.092*	0.323 (9)
H2D	−0.1030	0.6064	1.0746	0.092*	0.323 (9)
C3	0.0870 (6)	0.6513 (2)	1.0308 (4)	0.0596 (13)	
C4	0.1788 (6)	0.6446 (2)	0.9042 (4)	0.0522 (11)	
C5	0.0705 (5)	0.64895 (18)	0.7754 (4)	0.0391 (9)	
H5	0.0561	0.6893	0.7597	0.047*	
C6	0.1477 (5)	0.6264 (2)	0.6482 (4)	0.0444 (10)	
H6A	0.2535	0.6416	0.6425	0.053*	
H6B	0.1559	0.5858	0.6542	0.053*	
C7	0.0559 (5)	0.64180 (19)	0.5227 (4)	0.0442 (10)	
H7	0.0513	0.6829	0.5189	0.053*	
C8	−0.1146 (5)	0.62046 (18)	0.5220 (4)	0.0380 (9)	
C9	−0.1924 (5)	0.63960 (18)	0.6580 (4)	0.0443 (10)	
H9	−0.1918	0.6808	0.6543	0.053*	
C10	−0.0983 (5)	0.62477 (17)	0.7901 (3)	0.0428 (10)	
C11	−0.3706 (6)	0.6233 (2)	0.6593 (5)	0.0604 (13)	
H11A	−0.4033	0.6220	0.7523	0.073*	
H11B	−0.4300	0.6532	0.6154	0.073*	
C12	−0.4176 (6)	0.5680 (3)	0.5925 (5)	0.0669 (14)	
H12A	−0.5306	0.5674	0.5762	0.080*	
H12B	−0.3907	0.5370	0.6521	0.080*	
C13	−0.3319 (6)	0.5609 (2)	0.4582 (5)	0.0566 (12)	
H13	−0.3673	0.5271	0.4100	0.068*	
C14	−0.1555 (5)	0.55858 (18)	0.4915 (4)	0.0462 (10)	
H14	−0.1320	0.5340	0.5685	0.055*	
C15	−0.2106 (6)	0.6491 (2)	0.4093 (4)	0.0500 (11)	
C16	−0.3460 (6)	0.6124 (2)	0.3714 (5)	0.0575 (13)	
C17	−0.4607 (8)	0.6273 (3)	0.2837 (6)	0.091 (2)	
H17A	−0.4583	0.6625	0.2428	0.109*	
H17B	−0.5426	0.6025	0.2638	0.109*	
C18	0.2971 (7)	0.6944 (3)	0.9021 (5)	0.0727 (16)	
H18A	0.2405	0.7292	0.9077	0.087*	
H18B	0.3548	0.6935	0.8199	0.087*	
H18C	0.3692	0.6913	0.9774	0.087*	
C19	0.2760 (6)	0.5904 (2)	0.9127 (5)	0.0645 (14)	
H19A	0.3420	0.5913	0.9922	0.077*	
H19B	0.3405	0.5874	0.8346	0.077*	
H19C	0.2064	0.5587	0.9166	0.077*	
C20	−0.0951 (6)	0.5615 (2)	0.8243 (5)	0.0592 (13)	
H20A	−0.0239	0.5425	0.7655	0.071*	
H20B	−0.1990	0.5461	0.8125	0.071*	
H20C	−0.0604	0.5565	0.9161	0.071*	
C21	0.0542 (7)	0.5098 (2)	0.3887 (5)	0.0637 (14)	
C22	0.1285 (10)	0.4971 (3)	0.2562 (7)	0.106 (3)	
H22A	0.2276	0.4786	0.2717	0.128*	
H22B	0.1453	0.5316	0.2081	0.128*	
H22C	0.0599	0.4731	0.2041	0.128*	
O2'	0.3469 (4)	0.85034 (16)	0.0269 (3)	0.0586 (9)	
H2O'	0.3975	0.8477	−0.0424	0.070*	
O3'	0.6721 (5)	0.77771 (15)	−0.0252 (4)	0.0660 (10)	
O4'	0.5734 (4)	0.93369 (14)	−0.0081 (3)	0.0550 (9)	
O5'	0.4016 (6)	0.9800 (2)	0.1187 (4)	0.1003 (16)	
O1'	0.3864 (11)	0.8451 (4)	0.7516 (5)	0.090 (3)	0.829 (19)
C1'	0.6870 (10)	0.8126 (4)	0.5231 (7)	0.064 (2)	0.829 (19)
H1'1	0.7421	0.7807	0.4855	0.077*	0.829 (19)
H1'2	0.7649	0.8368	0.5661	0.077*	0.829 (19)
C2'	0.5725 (10)	0.7913 (5)	0.6304 (8)	0.072 (3)	0.829 (19)
H2'1	0.5420	0.7531	0.6076	0.087*	0.829 (19)
H2'2	0.6284	0.7900	0.7164	0.087*	0.829 (19)
O1*	0.350 (5)	0.818 (2)	0.746 (3)	0.090 (3)	0.171 (19)
C1*	0.666 (8)	0.806 (2)	0.521 (2)	0.064 (2)	0.171 (19)
H1*1	0.6369	0.7680	0.5005	0.077*	0.171 (19)
H1*2	0.7805	0.8085	0.5255	0.077*	0.171 (19)
C2*	0.599 (3)	0.8233 (17)	0.658 (2)	0.039 (8)	0.171 (19)
H2*1	0.6315	0.7962	0.7264	0.047*	0.171 (19)
H2*2	0.6387	0.8599	0.6848	0.047*	0.171 (19)
C3'	0.4290 (6)	0.8247 (2)	0.6466 (4)	0.0588 (13)	
C4'	0.3273 (6)	0.8314 (2)	0.5166 (4)	0.0476 (11)	
C5'	0.4329 (5)	0.82451 (17)	0.3899 (4)	0.0399 (9)	
H5'	0.4410	0.7839	0.3754	0.048*	
C6'	0.3526 (5)	0.84739 (19)	0.2639 (4)	0.0438 (10)	
H6'1	0.3479	0.8881	0.2694	0.053*	
H6'2	0.2454	0.8333	0.2583	0.053*	
C7'	0.4394 (5)	0.83058 (19)	0.1374 (4)	0.0416 (9)	
H7'	0.4407	0.7894	0.1331	0.050*	
C8'	0.6112 (5)	0.85099 (16)	0.1395 (4)	0.0373 (9)	
C9'	0.6931 (5)	0.83068 (19)	0.2744 (4)	0.0429 (10)	
H9'	0.6895	0.7895	0.2694	0.051*	
C10'	0.6046 (5)	0.84534 (16)	0.4075 (3)	0.0415 (10)	
C11'	0.8721 (5)	0.8451 (2)	0.2767 (5)	0.0597 (13)	
H11C	0.9092	0.8451	0.3697	0.072*	
H11D	0.9275	0.8154	0.2305	0.072*	
C12'	0.9178 (6)	0.9010 (3)	0.2136 (5)	0.0667 (14)	
H12C	0.8945	0.9313	0.2754	0.080*	
H12D	1.0302	0.9013	0.1977	0.080*	
C13'	0.8294 (6)	0.9105 (2)	0.0819 (5)	0.0585 (12)	
H13'	0.8646	0.9449	0.0370	0.070*	
C14'	0.6525 (5)	0.91288 (19)	0.1136 (4)	0.0459 (10)	
H14'	0.6315	0.9366	0.1919	0.055*	
C15'	0.7050 (6)	0.8232 (2)	0.0252 (4)	0.0501 (11)	
C16'	0.8382 (6)	0.8602 (2)	−0.0094 (5)	0.0587 (13)	
C17'	0.9457 (7)	0.8461 (3)	−0.0995 (6)	0.088 (2)	
H17C	0.9389	0.8117	−0.1437	0.106*	
H17D	1.0277	0.8707	−0.1183	0.106*	
C18'	0.2105 (7)	0.7819 (3)	0.5205 (6)	0.0732 (16)	
H18D	0.1380	0.7875	0.5924	0.088*	
H18E	0.2674	0.7475	0.5350	0.088*	
H18F	0.1532	0.7798	0.4363	0.088*	
C19'	0.2339 (6)	0.8872 (2)	0.5239 (5)	0.0613 (13)	
H19D	0.1804	0.8892	0.6081	0.074*	
H19E	0.1581	0.8887	0.4510	0.074*	
H19F	0.3054	0.9183	0.5170	0.074*	
C20'	0.6079 (6)	0.9084 (2)	0.4436 (5)	0.0559 (12)	
H20D	0.5455	0.9290	0.3788	0.067*	
H20E	0.7146	0.9217	0.4427	0.067*	
H20F	0.5657	0.9137	0.5317	0.067*	
C21'	0.4459 (7)	0.9658 (2)	0.0084 (5)	0.0643 (14)	
C22'	0.3709 (9)	0.9785 (3)	−0.1242 (7)	0.095 (2)	
H22D	0.2715	0.9594	−0.1315	0.114*	
H22E	0.4384	0.9661	−0.1948	0.114*	
H22F	0.3542	1.0183	−0.1320	0.114*	

### Atomic displacement parameters (Å^2^)

**Table:**

	*U*^11^	*U*^22^	*U*^33^	*U*^12^	*U*^13^	*U*^23^
O2	0.053 (2)	0.099 (3)	0.0356 (15)	−0.0010 (19)	0.0084 (15)	0.0041 (16)
O3	0.087 (3)	0.059 (2)	0.057 (2)	0.0069 (19)	−0.0092 (19)	0.0131 (17)
O4	0.063 (2)	0.0525 (18)	0.0430 (16)	0.0125 (16)	−0.0033 (15)	−0.0103 (13)
O5	0.112 (4)	0.113 (4)	0.076 (3)	0.068 (3)	0.006 (3)	0.016 (2)
O1	0.120 (4)	0.138 (8)	0.033 (2)	0.059 (6)	0.007 (2)	0.027 (4)
C1	0.051 (3)	0.097 (5)	0.046 (3)	0.013 (3)	0.007 (2)	−0.016 (3)
C2	0.095 (6)	0.097 (8)	0.038 (4)	0.032 (7)	0.001 (4)	−0.015 (5)
O1"	0.120 (4)	0.138 (8)	0.033 (2)	0.059 (6)	0.007 (2)	0.027 (4)
C1"	0.051 (3)	0.097 (5)	0.046 (3)	0.013 (3)	0.007 (2)	−0.016 (3)
C2"	0.095 (6)	0.097 (8)	0.038 (4)	0.032 (7)	0.001 (4)	−0.015 (5)
C3	0.069 (4)	0.076 (3)	0.033 (2)	0.003 (3)	−0.006 (2)	0.000 (2)
C4	0.056 (3)	0.064 (3)	0.037 (2)	−0.004 (2)	0.002 (2)	0.002 (2)
C5	0.045 (2)	0.038 (2)	0.0333 (18)	−0.0027 (18)	−0.0041 (17)	0.0023 (16)
C6	0.037 (2)	0.061 (3)	0.036 (2)	−0.006 (2)	0.0068 (18)	0.0014 (19)
C7	0.050 (3)	0.053 (2)	0.0302 (18)	0.002 (2)	0.0048 (18)	0.0022 (17)
C8	0.038 (2)	0.043 (2)	0.0332 (18)	0.0036 (18)	−0.0027 (17)	−0.0001 (16)
C9	0.041 (2)	0.046 (2)	0.046 (2)	0.0038 (19)	0.0025 (19)	−0.0091 (18)
C10	0.044 (2)	0.047 (2)	0.037 (2)	−0.001 (2)	0.0069 (18)	0.0002 (18)
C11	0.037 (3)	0.088 (4)	0.056 (3)	0.006 (3)	0.005 (2)	−0.015 (3)
C12	0.044 (3)	0.088 (4)	0.069 (3)	−0.018 (3)	0.003 (2)	−0.013 (3)
C13	0.049 (3)	0.060 (3)	0.061 (3)	−0.007 (2)	−0.005 (2)	−0.016 (2)
C14	0.054 (3)	0.042 (2)	0.042 (2)	0.001 (2)	0.003 (2)	−0.0048 (18)
C15	0.059 (3)	0.052 (3)	0.039 (2)	0.015 (2)	−0.001 (2)	−0.003 (2)
C16	0.048 (3)	0.068 (3)	0.057 (3)	0.011 (2)	−0.009 (2)	−0.011 (2)
C17	0.068 (4)	0.103 (5)	0.100 (5)	0.016 (4)	−0.034 (4)	−0.010 (4)
C18	0.065 (4)	0.098 (4)	0.055 (3)	−0.025 (3)	−0.010 (3)	−0.003 (3)
C19	0.055 (3)	0.088 (4)	0.051 (3)	0.011 (3)	−0.012 (2)	0.011 (2)
C20	0.059 (3)	0.066 (3)	0.052 (3)	−0.015 (2)	−0.001 (2)	0.015 (2)
C21	0.070 (4)	0.063 (3)	0.059 (3)	0.021 (3)	0.004 (3)	−0.006 (2)
C22	0.128 (7)	0.109 (5)	0.084 (4)	0.039 (5)	0.029 (4)	−0.035 (4)
O2'	0.0475 (19)	0.096 (3)	0.0321 (14)	0.0080 (18)	−0.0098 (13)	0.0048 (16)
O3'	0.075 (3)	0.063 (2)	0.060 (2)	0.0144 (19)	0.0030 (18)	−0.0161 (17)
O4'	0.063 (2)	0.0590 (19)	0.0433 (16)	0.0187 (17)	0.0021 (15)	0.0113 (14)
O5'	0.121 (4)	0.108 (3)	0.072 (3)	0.068 (3)	−0.003 (3)	−0.019 (2)
O1'	0.115 (5)	0.125 (7)	0.0296 (18)	0.023 (5)	−0.004 (2)	−0.004 (3)
C1'	0.062 (5)	0.087 (4)	0.043 (2)	0.012 (4)	−0.013 (2)	0.010 (3)
C2'	0.084 (6)	0.085 (7)	0.048 (4)	0.009 (5)	−0.009 (4)	0.025 (4)
O1*	0.115 (5)	0.125 (7)	0.0296 (18)	0.023 (5)	−0.004 (2)	−0.004 (3)
C1*	0.062 (5)	0.087 (4)	0.043 (2)	0.012 (4)	−0.013 (2)	0.010 (3)
C2*	0.046 (16)	0.046 (19)	0.025 (11)	−0.016 (13)	−0.019 (10)	−0.005 (12)
C3'	0.075 (4)	0.067 (3)	0.033 (2)	−0.012 (3)	−0.002 (2)	0.005 (2)
C4'	0.053 (3)	0.054 (3)	0.035 (2)	−0.003 (2)	−0.0004 (19)	0.0050 (19)
C5'	0.047 (2)	0.042 (2)	0.0309 (18)	−0.0028 (18)	−0.0043 (17)	0.0004 (16)
C6'	0.040 (2)	0.056 (3)	0.035 (2)	−0.003 (2)	−0.0070 (18)	0.0007 (19)
C7'	0.041 (2)	0.052 (2)	0.0312 (19)	0.000 (2)	−0.0090 (17)	−0.0043 (18)
C8'	0.038 (2)	0.039 (2)	0.0343 (19)	0.0083 (18)	−0.0045 (17)	0.0039 (16)
C9'	0.041 (2)	0.047 (2)	0.041 (2)	0.0046 (19)	−0.0070 (18)	0.0029 (18)
C10'	0.043 (2)	0.047 (2)	0.034 (2)	−0.0011 (19)	−0.0075 (17)	0.0014 (17)
C11'	0.040 (3)	0.082 (3)	0.057 (3)	0.010 (3)	−0.010 (2)	0.004 (2)
C12'	0.041 (3)	0.092 (4)	0.067 (3)	−0.010 (3)	0.001 (2)	0.004 (3)
C13'	0.058 (3)	0.059 (3)	0.059 (3)	−0.001 (2)	0.006 (2)	0.007 (2)
C14'	0.047 (3)	0.049 (2)	0.041 (2)	0.004 (2)	−0.004 (2)	0.0032 (19)
C15'	0.047 (3)	0.062 (3)	0.041 (2)	0.014 (2)	−0.002 (2)	0.006 (2)
C16'	0.052 (3)	0.079 (3)	0.045 (2)	0.014 (3)	0.004 (2)	0.011 (2)
C17'	0.071 (4)	0.115 (5)	0.080 (4)	0.029 (4)	0.029 (3)	0.008 (4)
C18'	0.078 (4)	0.084 (4)	0.057 (3)	−0.028 (3)	0.006 (3)	0.009 (3)
C19'	0.056 (3)	0.076 (3)	0.052 (3)	0.013 (3)	0.007 (2)	0.001 (2)
C20'	0.059 (3)	0.055 (3)	0.054 (3)	−0.012 (2)	−0.001 (2)	−0.013 (2)
C21'	0.075 (4)	0.051 (3)	0.066 (3)	0.024 (3)	−0.006 (3)	0.006 (2)
C22'	0.092 (5)	0.100 (5)	0.091 (4)	0.042 (4)	−0.018 (4)	0.027 (4)

### Geometric parameters (Å, °)

**Table:**

O2—C7	1.416 (5)	O2'—C7'	1.417 (5)
O2—H2O	0.8200	O2'—H2O'	0.8200
O3—C15	1.218 (6)	O3'—C15'	1.223 (6)
O4—C21	1.344 (6)	O4'—C21'	1.337 (6)
O4—C14	1.447 (5)	O4'—C14'	1.458 (5)
O5—C21	1.195 (6)	O5'—C21'	1.213 (6)
O1—C3	1.212 (3)	O1'—C3'	1.211 (3)
C1—C2	1.540 (3)	C1'—C2'	1.540 (3)
C1—C10	1.543 (3)	C1'—C10'	1.543 (3)
C1—H1A	0.9700	C1'—H1'1	0.9700
C1—H1B	0.9700	C1'—H1'2	0.9700
C2—C3	1.488 (11)	C2'—C3'	1.464 (3)
C2—H2A	0.9700	C2'—H2'1	0.9700
C2—H2B	0.9700	C2'—H2'2	0.9700
O1"—C3	1.212 (3)	O1*—C3'	1.210 (3)
C1"—C2"	1.540 (3)	C1*—C2*	1.540 (3)
C1"—C10	1.541 (3)	C1*—C10'	1.541 (3)
C1"—H1C	0.9700	C1*—H1*1	0.9700
C1"—H1D	0.9700	C1*—H1*2	0.9700
C2"—C3	1.45 (3)	C2*—C3'	1.45 (3)
C2"—H2C	0.9700	C2*—H2*1	0.9700
C2"—H2D	0.9700	C2*—H2*2	0.9700
C3—C4	1.499 (6)	C3'—C4'	1.548 (6)
C4—C19	1.532 (7)	C4'—C18'	1.541 (7)
C4—C18	1.553 (7)	C4'—C19'	1.548 (7)
C4—C5	1.565 (6)	C4'—C5'	1.567 (6)
C5—C6	1.530 (5)	C5'—C6'	1.515 (5)
C5—C10	1.553 (6)	C5'—C10'	1.545 (6)
C5—H5	0.9800	C5'—H5'	0.9800
C6—C7	1.502 (6)	C6'—C7'	1.521 (5)
C6—H6A	0.9700	C6'—H6'1	0.9700
C6—H6B	0.9700	C6'—H6'2	0.9700
C7—C8	1.533 (6)	C7'—C8'	1.536 (6)
C7—H7	0.9800	C7'—H7'	0.9800
C8—C15	1.533 (6)	C8'—C14'	1.536 (6)
C8—C14	1.541 (6)	C8'—C15'	1.546 (6)
C8—C9	1.580 (5)	C8'—C9'	1.574 (5)
C9—C11	1.562 (6)	C9'—C11'	1.558 (6)
C9—C10	1.563 (6)	C9'—C10'	1.571 (5)
C9—H9	0.9800	C9'—H9'	0.9800
C10—C20	1.543 (7)	C10'—C20'	1.542 (6)
C11—C12	1.524 (8)	C11'—C12'	1.521 (8)
C11—H11A	0.9700	C11'—H11C	0.9700
C11—H11B	0.9700	C11'—H11D	0.9700
C12—C13	1.540 (7)	C12'—C13'	1.512 (8)
C12—H12A	0.9700	C12'—H12C	0.9700
C12—H12B	0.9700	C12'—H12D	0.9700
C13—C16	1.502 (7)	C13'—C16'	1.504 (7)
C13—C14	1.528 (7)	C13'—C14'	1.540 (7)
C13—H13	0.9800	C13'—H13'	0.9800
C14—H14	0.9800	C14'—H14'	0.9800
C15—C16	1.487 (7)	C15'—C16'	1.478 (7)
C16—C17	1.342 (8)	C16'—C17'	1.332 (7)
C17—H17A	0.9300	C17'—H17C	0.9300
C17—H17B	0.9300	C17'—H17D	0.9300
C18—H18A	0.9600	C18'—H18D	0.9600
C18—H18B	0.9600	C18'—H18E	0.9600
C18—H18C	0.9600	C18'—H18F	0.9600
C19—H19A	0.9600	C19'—H19D	0.9600
C19—H19B	0.9600	C19'—H19E	0.9600
C19—H19C	0.9600	C19'—H19F	0.9600
C20—H20A	0.9600	C20'—H20D	0.9600
C20—H20B	0.9600	C20'—H20E	0.9600
C20—H20C	0.9600	C20'—H20F	0.9600
C21—C22	1.499 (8)	C21'—C22'	1.482 (8)
C22—H22A	0.9600	C22'—H22D	0.9600
C22—H22B	0.9600	C22'—H22E	0.9600
C22—H22C	0.9600	C22'—H22F	0.9600
			
C7—O2—H2O	109.5	C7'—O2'—H2O'	109.5
C21—O4—C14	116.2 (4)	C21'—O4'—C14'	117.0 (4)
C2—C1—C10	115.1 (7)	C2'—C1'—C10'	113.4 (5)
C2—C1—H1A	108.5	C2'—C1'—H1'1	108.9
C10—C1—H1A	108.5	C10'—C1'—H1'1	108.9
C2—C1—H1B	108.5	C2'—C1'—H1'2	108.9
C10—C1—H1B	108.5	C10'—C1'—H1'2	108.9
H1A—C1—H1B	107.5	H1'1—C1'—H1'2	107.7
C3—C2—C1	116.7 (7)	C3'—C2'—C1'	115.7 (5)
C3—C2—H2A	108.1	C3'—C2'—H2'1	108.4
C1—C2—H2A	108.1	C1'—C2'—H2'1	108.4
C3—C2—H2B	108.1	C3'—C2'—H2'2	108.4
C1—C2—H2B	108.1	C1'—C2'—H2'2	108.4
H2A—C2—H2B	107.3	H2'1—C2'—H2'2	107.4
C2"—C1"—C10	112.6 (12)	C2*—C1*—C10'	111.4 (16)
C2"—C1"—H1C	109.1	C2*—C1*—H1*1	109.3
C10—C1"—H1C	109.1	C10'—C1*—H1*1	109.3
C2"—C1"—H1D	109.1	C2*—C1*—H1*2	109.3
C10—C1"—H1D	109.1	C10'—C1*—H1*2	109.3
H1C—C1"—H1D	107.8	H1*1—C1*—H1*2	108.0
C3—C2"—C1"	112.7 (19)	C3'—C2*—C1*	109 (3)
C3—C2"—H2C	109.0	C3'—C2*—H2*1	110.0
C1"—C2"—H2C	109.0	C1*—C2*—H2*1	110.0
C3—C2"—H2D	109.0	C3'—C2*—H2*2	110.0
C1"—C2"—H2D	109.0	C1*—C2*—H2*2	110.0
H2C—C2"—H2D	107.8	H2*1—C2*—H2*2	108.3
O1"—C3—O1	43.4 (7)	O1*—C3'—O1'	34 (2)
O1"—C3—C2"	112.3 (12)	O1*—C3'—C2*	120 (2)
O1—C3—C2"	102.0 (10)	O1'—C3'—C2*	104.7 (12)
O1"—C3—C2	106.7 (9)	O1*—C3'—C2'	120 (2)
O1—C3—C2	123.3 (6)	O1'—C3'—C2'	125.0 (7)
C2"—C3—C2	35.4 (9)	C2*—C3'—C2'	33.5 (12)
O1"—C3—C4	121.0 (9)	O1*—C3'—C4'	113 (2)
O1—C3—C4	120.7 (6)	O1'—C3'—C4'	120.4 (5)
C2"—C3—C4	126.3 (8)	C2*—C3'—C4'	127.6 (10)
C2—C3—C4	115.9 (5)	C2'—C3'—C4'	114.6 (5)
C3—C4—C19	109.3 (4)	C18'—C4'—C19'	108.9 (4)
C3—C4—C18	106.0 (4)	C18'—C4'—C3'	104.4 (4)
C19—C4—C18	107.2 (4)	C19'—C4'—C3'	109.2 (4)
C3—C4—C5	111.8 (4)	C18'—C4'—C5'	108.5 (4)
C19—C4—C5	114.1 (4)	C19'—C4'—C5'	115.3 (3)
C18—C4—C5	108.0 (4)	C3'—C4'—C5'	109.9 (4)
C6—C5—C10	110.9 (3)	C6'—C5'—C10'	112.8 (3)
C6—C5—C4	113.5 (4)	C6'—C5'—C4'	111.8 (4)
C10—C5—C4	115.4 (3)	C10'—C5'—C4'	115.3 (3)
C6—C5—H5	105.4	C6'—C5'—H5'	105.3
C10—C5—H5	105.4	C10'—C5'—H5'	105.3
C4—C5—H5	105.4	C4'—C5'—H5'	105.3
C7—C6—C5	112.1 (4)	C5'—C6'—C7'	111.8 (4)
C7—C6—H6A	109.2	C5'—C6'—H6'1	109.3
C5—C6—H6A	109.2	C7'—C6'—H6'1	109.3
C7—C6—H6B	109.2	C5'—C6'—H6'2	109.3
C5—C6—H6B	109.2	C7'—C6'—H6'2	109.3
H6A—C6—H6B	107.9	H6'1—C6'—H6'2	107.9
O2—C7—C6	106.6 (4)	O2'—C7'—C6'	106.4 (3)
O2—C7—C8	115.1 (4)	O2'—C7'—C8'	114.7 (3)
C6—C7—C8	113.5 (3)	C6'—C7'—C8'	112.3 (3)
O2—C7—H7	107.1	O2'—C7'—H7'	107.7
C6—C7—H7	107.1	C6'—C7'—H7'	107.7
C8—C7—H7	107.1	C8'—C7'—H7'	107.7
C15—C8—C7	110.2 (4)	C14'—C8'—C7'	121.3 (3)
C15—C8—C14	99.6 (3)	C14'—C8'—C15'	99.5 (3)
C7—C8—C14	121.8 (4)	C7'—C8'—C15'	110.9 (3)
C15—C8—C9	105.7 (3)	C14'—C8'—C9'	109.8 (3)
C7—C8—C9	108.0 (3)	C7'—C8'—C9'	108.6 (3)
C14—C8—C9	110.3 (3)	C15'—C8'—C9'	105.6 (3)
C11—C9—C10	114.7 (4)	C11'—C9'—C10'	114.8 (4)
C11—C9—C8	110.8 (3)	C11'—C9'—C8'	111.1 (3)
C10—C9—C8	115.9 (3)	C10'—C9'—C8'	116.0 (3)
C11—C9—H9	104.7	C11'—C9'—H9'	104.5
C10—C9—H9	104.7	C10'—C9'—H9'	104.5
C8—C9—H9	104.7	C8'—C9'—H9'	104.5
C1"—C10—C20	106.6 (18)	C1*—C10'—C20'	114 (2)
C1"—C10—C1	4.2 (17)	C1*—C10'—C1'	8(3)
C20—C10—C1	109.7 (9)	C20'—C10'—C1'	108.2 (5)
C1"—C10—C5	107.1 (16)	C1*—C10'—C5'	101 (3)
C20—C10—C5	111.7 (4)	C20'—C10'—C5'	110.6 (4)
C1—C10—C5	108.0 (7)	C1'—C10'—C5'	109.6 (5)
C1"—C10—C9	109.7 (9)	C1*—C10'—C9'	108.7 (12)
C20—C10—C9	114.3 (4)	C20'—C10'—C9'	113.8 (3)
C1—C10—C9	105.6 (6)	C1'—C10'—C9'	107.2 (4)
C5—C10—C9	107.2 (3)	C5'—C10'—C9'	107.3 (3)
C12—C11—C9	117.1 (4)	C12'—C11'—C9'	116.3 (4)
C12—C11—H11A	108.0	C12'—C11'—H11C	108.2
C9—C11—H11A	108.0	C9'—C11'—H11C	108.2
C12—C11—H11B	108.0	C12'—C11'—H11D	108.2
C9—C11—H11B	108.0	C9'—C11'—H11D	108.2
H11A—C11—H11B	107.3	H11C—C11'—H11D	107.4
C11—C12—C13	110.3 (4)	C13'—C12'—C11'	111.2 (4)
C11—C12—H12A	109.6	C13'—C12'—H12C	109.4
C13—C12—H12A	109.6	C11'—C12'—H12C	109.4
C11—C12—H12B	109.6	C13'—C12'—H12D	109.4
C13—C12—H12B	109.6	C11'—C12'—H12D	109.4
H12A—C12—H12B	108.1	H12C—C12'—H12D	108.0
C16—C13—C14	102.8 (4)	C16'—C13'—C12'	111.9 (4)
C16—C13—C12	112.0 (4)	C16'—C13'—C14'	102.2 (4)
C14—C13—C12	107.0 (4)	C12'—C13'—C14'	107.4 (4)
C16—C13—H13	111.5	C16'—C13'—H13'	111.6
C14—C13—H13	111.5	C12'—C13'—H13'	111.6
C12—C13—H13	111.5	C14'—C13'—H13'	111.6
O4—C14—C13	106.6 (4)	O4'—C14'—C8'	111.2 (4)
O4—C14—C8	110.8 (3)	O4'—C14'—C13'	106.2 (3)
C13—C14—C8	103.0 (4)	C8'—C14'—C13'	103.0 (3)
O4—C14—H14	112.0	O4'—C14'—H14'	112.0
C13—C14—H14	112.0	C8'—C14'—H14'	112.0
C8—C14—H14	112.0	C13'—C14'—H14'	112.0
O3—C15—C16	125.9 (5)	O3'—C15'—C16'	127.0 (4)
O3—C15—C8	125.3 (5)	O3'—C15'—C8'	124.2 (4)
C16—C15—C8	108.7 (4)	C16'—C15'—C8'	108.8 (4)
C17—C16—C15	123.8 (5)	C17'—C16'—C15'	122.9 (6)
C17—C16—C13	129.7 (6)	C17'—C16'—C13'	130.3 (6)
C15—C16—C13	106.3 (4)	C15'—C16'—C13'	106.7 (4)
C16—C17—H17A	120.0	C16'—C17'—H17C	120.0
C16—C17—H17B	120.0	C16'—C17'—H17D	120.0
H17A—C17—H17B	120.0	H17C—C17'—H17D	120.0
C4—C18—H18A	109.5	C4'—C18'—H18D	109.5
C4—C18—H18B	109.5	C4'—C18'—H18E	109.5
H18A—C18—H18B	109.5	H18D—C18'—H18E	109.5
C4—C18—H18C	109.5	C4'—C18'—H18F	109.5
H18A—C18—H18C	109.5	H18D—C18'—H18F	109.5
H18B—C18—H18C	109.5	H18E—C18'—H18F	109.5
C4—C19—H19A	109.5	C4'—C19'—H19D	109.5
C4—C19—H19B	109.5	C4'—C19'—H19E	109.5
H19A—C19—H19B	109.5	H19D—C19'—H19E	109.5
C4—C19—H19C	109.5	C4'—C19'—H19F	109.5
H19A—C19—H19C	109.5	H19D—C19'—H19F	109.5
H19B—C19—H19C	109.5	H19E—C19'—H19F	109.5
C10—C20—H20A	109.5	C10'—C20'—H20D	109.5
C10—C20—H20B	109.5	C10'—C20'—H20E	109.5
H20A—C20—H20B	109.5	H20D—C20'—H20E	109.5
C10—C20—H20C	109.5	C10'—C20'—H20F	109.5
H20A—C20—H20C	109.5	H20D—C20'—H20F	109.5
H20B—C20—H20C	109.5	H20E—C20'—H20F	109.5
O5—C21—O4	123.1 (5)	O5'—C21'—O4'	122.3 (5)
O5—C21—C22	126.1 (6)	O5'—C21'—C22'	127.6 (5)
O4—C21—C22	110.7 (5)	O4'—C21'—C22'	110.1 (5)
C21—C22—H22A	109.5	C21'—C22'—H22D	109.5
C21—C22—H22B	109.5	C21'—C22'—H22E	109.5
H22A—C22—H22B	109.5	H22D—C22'—H22E	109.5
C21—C22—H22C	109.5	C21'—C22'—H22F	109.5
H22A—C22—H22C	109.5	H22D—C22'—H22F	109.5
H22B—C22—H22C	109.5	H22E—C22'—H22F	109.5
			
C10—C1—C2—C3	−21 (2)	C10'—C1*—C2*—C3'	54 (6)
C10—C1"—C2"—C3	47 (4)	C1*—C2*—C3'—O1*	156 (4)
C1"—C2"—C3—O1"	147 (2)	C1*—C2*—C3'—O1'	−171 (3)
C1"—C2"—C3—O1	−169 (2)	C1*—C2*—C3'—C2'	56 (3)
C1"—C2"—C3—C2	59 (2)	C1*—C2*—C3'—C4'	−22 (4)
C1"—C2"—C3—C4	−26 (3)	C1'—C2'—C3'—O1*	−164 (3)
C1—C2—C3—O1"	−172.2 (14)	C1'—C2'—C3'—O1'	−124.4 (13)
C1—C2—C3—O1	−127.4 (14)	C1'—C2'—C3'—C2*	−64 (2)
C1—C2—C3—C2"	−67 (2)	C1'—C2'—C3'—C4'	57.3 (12)
C1—C2—C3—C4	49.8 (13)	O1*—C3'—C4'—C18'	−50 (3)
O1"—C3—C4—C19	80.5 (11)	O1'—C3'—C4'—C18'	−87.0 (8)
O1—C3—C4—C19	29.5 (9)	C2*—C3'—C4'—C18'	128 (2)
C2"—C3—C4—C19	−107.9 (14)	C2'—C3'—C4'—C18'	91.3 (7)
C2—C3—C4—C19	−147.8 (7)	O1*—C3'—C4'—C19'	66 (3)
O1"—C3—C4—C18	−34.7 (11)	O1'—C3'—C4'—C19'	29.4 (9)
O1—C3—C4—C18	−85.8 (8)	C2*—C3'—C4'—C19'	−116 (2)
C2"—C3—C4—C18	136.9 (14)	C2'—C3'—C4'—C19'	−152.3 (6)
C2—C3—C4—C18	97.0 (7)	O1*—C3'—C4'—C5'	−166 (3)
O1"—C3—C4—C5	−152.2 (10)	O1'—C3'—C4'—C5'	156.8 (8)
O1—C3—C4—C5	156.8 (7)	C2*—C3'—C4'—C5'	12 (2)
C2"—C3—C4—C5	19.4 (15)	C2'—C3'—C4'—C5'	−24.8 (7)
C2—C3—C4—C5	−20.5 (8)	C18'—C4'—C5'—C6'	83.3 (5)
C3—C4—C5—C6	−163.8 (4)	C19'—C4'—C5'—C6'	−39.1 (5)
C19—C4—C5—C6	−39.2 (5)	C3'—C4'—C5'—C6'	−163.1 (4)
C18—C4—C5—C6	79.9 (5)	C18'—C4'—C5'—C10'	−146.2 (4)
C3—C4—C5—C10	−34.3 (5)	C19'—C4'—C5'—C10'	91.3 (5)
C19—C4—C5—C10	90.3 (5)	C3'—C4'—C5'—C10'	−32.6 (5)
C18—C4—C5—C10	−150.6 (4)	C10'—C5'—C6'—C7'	59.5 (5)
C10—C5—C6—C7	60.0 (5)	C4'—C5'—C6'—C7'	−168.8 (4)
C4—C5—C6—C7	−168.2 (4)	C5'—C6'—C7'—O2'	175.4 (3)
C5—C6—C7—O2	174.2 (3)	C5'—C6'—C7'—C8'	−58.4 (5)
C5—C6—C7—C8	−58.2 (5)	O2'—C7'—C8'—C14'	45.9 (5)
O2—C7—C8—C15	−70.4 (5)	C6'—C7'—C8'—C14'	−75.7 (5)
C6—C7—C8—C15	166.5 (3)	O2'—C7'—C8'—C15'	−70.0 (4)
O2—C7—C8—C14	45.5 (5)	C6'—C7'—C8'—C15'	168.3 (4)
C6—C7—C8—C14	−77.6 (5)	O2'—C7'—C8'—C9'	174.4 (3)
O2—C7—C8—C9	174.6 (3)	C6'—C7'—C8'—C9'	52.8 (4)
C6—C7—C8—C9	51.5 (5)	C14'—C8'—C9'—C11'	−50.7 (4)
C15—C8—C9—C11	57.6 (5)	C7'—C8'—C9'—C11'	174.6 (4)
C7—C8—C9—C11	175.5 (4)	C15'—C8'—C9'—C11'	55.7 (5)
C14—C8—C9—C11	−49.2 (5)	C14'—C8'—C9'—C10'	82.8 (4)
C15—C8—C9—C10	−169.5 (4)	C7'—C8'—C9'—C10'	−51.9 (4)
C7—C8—C9—C10	−51.6 (5)	C15'—C8'—C9'—C10'	−170.8 (4)
C14—C8—C9—C10	83.7 (4)	C2*—C1*—C10'—C20'	45 (6)
C2"—C1"—C10—C20	58 (3)	C2*—C1*—C10'—C1'	92 (11)
C2"—C1"—C10—C5	−61 (3)	C2*—C1*—C10'—C5'	−74 (5)
C2"—C1"—C10—C9	−177 (2)	C2*—C1*—C10'—C9'	173 (4)
C2—C1—C10—C20	91.6 (15)	C2'—C1'—C10'—C1*	−43 (9)
C2—C1—C10—C5	−30.3 (17)	C2'—C1'—C10'—C20'	92.9 (9)
C2—C1—C10—C9	−144.8 (13)	C2'—C1'—C10'—C5'	−27.7 (10)
C6—C5—C10—C1"	−173.6 (14)	C2'—C1'—C10'—C9'	−143.9 (8)
C4—C5—C10—C1"	55.6 (14)	C6'—C5'—C10'—C1*	−168.0 (14)
C6—C5—C10—C20	70.0 (4)	C4'—C5'—C10'—C1*	62.0 (14)
C4—C5—C10—C20	−60.8 (4)	C6'—C5'—C10'—C20'	70.6 (4)
C6—C5—C10—C1	−169.3 (8)	C4'—C5'—C10'—C20'	−59.4 (4)
C4—C5—C10—C1	59.9 (9)	C6'—C5'—C10'—C1'	−170.3 (5)
C6—C5—C10—C9	−55.9 (4)	C4'—C5'—C10'—C1'	59.7 (6)
C4—C5—C10—C9	173.3 (3)	C6'—C5'—C10'—C9'	−54.1 (4)
C11—C9—C10—C1"	−59 (2)	C4'—C5'—C10'—C9'	175.9 (3)
C8—C9—C10—C1"	170 (2)	C11'—C9'—C10'—C1*	−67 (3)
C11—C9—C10—C20	61.0 (5)	C8'—C9'—C10'—C1*	161 (3)
C8—C9—C10—C20	−70.1 (5)	C11'—C9'—C10'—C20'	61.1 (5)
C11—C9—C10—C1	−59.7 (10)	C8'—C9'—C10'—C20'	−70.7 (5)
C8—C9—C10—C1	169.2 (9)	C11'—C9'—C10'—C1'	−58.6 (6)
C11—C9—C10—C5	−174.7 (4)	C8'—C9'—C10'—C1'	169.7 (6)
C8—C9—C10—C5	54.2 (4)	C11'—C9'—C10'—C5'	−176.3 (4)
C10—C9—C11—C12	−97.5 (5)	C8'—C9'—C10'—C5'	52.0 (4)
C8—C9—C11—C12	36.1 (6)	C10'—C9'—C11'—C12'	−96.8 (5)
C9—C11—C12—C13	−42.9 (7)	C8'—C9'—C11'—C12'	37.2 (6)
C11—C12—C13—C16	−49.3 (6)	C9'—C11'—C12'—C13'	−43.4 (6)
C11—C12—C13—C14	62.6 (6)	C11'—C12'—C13'—C16'	−49.3 (6)
C21—O4—C14—C13	−149.3 (4)	C11'—C12'—C13'—C14'	62.1 (5)
C21—O4—C14—C8	99.4 (5)	C21'—O4'—C14'—C8'	102.2 (5)
C16—C13—C14—O4	−74.1 (4)	C21'—O4'—C14'—C13'	−146.4 (4)
C12—C13—C14—O4	167.8 (4)	C7'—C8'—C14'—O4'	−49.8 (5)
C16—C13—C14—C8	42.5 (4)	C15'—C8'—C14'—O4'	71.8 (4)
C12—C13—C14—C8	−75.6 (4)	C9'—C8'—C14'—O4'	−177.8 (3)
C15—C8—C14—O4	71.9 (4)	C7'—C8'—C14'—C13'	−163.2 (4)
C7—C8—C14—O4	−49.2 (5)	C15'—C8'—C14'—C13'	−41.6 (4)
C9—C8—C14—O4	−177.3 (3)	C9'—C8'—C14'—C13'	68.9 (4)
C15—C8—C14—C13	−41.7 (4)	C16'—C13'—C14'—O4'	−73.8 (4)
C7—C8—C14—C13	−162.8 (4)	C12'—C13'—C14'—O4'	168.3 (4)
C9—C8—C14—C13	69.1 (4)	C16'—C13'—C14'—C8'	43.1 (4)
C7—C8—C15—O3	−24.0 (6)	C12'—C13'—C14'—C8'	−74.7 (4)
C14—C8—C15—O3	−153.2 (4)	C14'—C8'—C15'—O3'	−154.8 (5)
C9—C8—C15—O3	92.4 (5)	C7'—C8'—C15'—O3'	−26.0 (6)
C7—C8—C15—C16	155.7 (3)	C9'—C8'—C15'—O3'	91.4 (5)
C14—C8—C15—C16	26.5 (4)	C14'—C8'—C15'—C16'	25.8 (4)
C9—C8—C15—C16	−87.9 (4)	C7'—C8'—C15'—C16'	154.7 (4)
O3—C15—C16—C17	−6.3 (8)	C9'—C8'—C15'—C16'	−87.9 (4)
C8—C15—C16—C17	174.1 (5)	O3'—C15'—C16'—C17'	−3.1 (8)
O3—C15—C16—C13	178.7 (4)	C8'—C15'—C16'—C17'	176.2 (5)
C8—C15—C16—C13	−1.0 (5)	O3'—C15'—C16'—C13'	−179.1 (5)
C14—C13—C16—C17	159.8 (5)	C8'—C15'—C16'—C13'	0.3 (5)
C12—C13—C16—C17	−85.6 (7)	C12'—C13'—C16'—C17'	−87.3 (7)
C14—C13—C16—C15	−25.4 (4)	C14'—C13'—C16'—C17'	158.1 (6)
C12—C13—C16—C15	89.1 (5)	C12'—C13'—C16'—C15'	88.2 (5)
C14—O4—C21—O5	6.5 (8)	C14'—C13'—C16'—C15'	−26.4 (5)
C14—O4—C21—C22	−175.3 (5)	C14'—O4'—C21'—O5'	4.0 (8)
C10'—C1'—C2'—C3'	−28.0 (14)	C14'—O4'—C21'—C22'	−174.3 (5)

### Hydrogen-bond geometry (Å, °)

**Table:**

*D*—H···*A*	*D*—H	H···*A*	*D*···*A*	*D*—H···*A*
O2—H2O···O1^i^	0.82	2.09	2.747 (6)	137
O2—H2O···O1^i^	0.82	2.09	2.747 (6)	137
O2'—H2O'···O1'^i^	0.82	2.05	2.764 (5)	146
O2'—H2O'···O1*^i^	0.82	2.24	2.89 (3)	136

Symmetry codes: (i) *x*, *y*, *z*−1.
